# Relationship of 24-hour ambulatory blood pressure and heart rate with markers of hepatic function in cirrhotic patients

**DOI:** 10.1186/1471-230X-10-143

**Published:** 2010-12-12

**Authors:** Dimitris G Tzamouranis, Alexandra Alexopoulou, Spyros P Dourakis, George S Stergiou

**Affiliations:** 1 Hypertension Center, 3rd Department of Medicine, University of Athens Medical School, Sotiria General Hospital, 152 Mesogeion Avenue, 11527, Athens, Greece; 2 2nd Department of Medicine, University of Athens Medical School, Hippokration General Hospital, 114 Vas. Sofias Avenue, 11527, Athens, Greece

## Abstract

**Background:**

There is evidence that in cirrhotic patients, certain hemodynamic parameters, such as blood pressure and heart rate, are related to the severity of liver disease. This study investigated whether non-invasive 24-hour ambulatory blood pressure and heart rate are more closely associated with markers of liver disease severity than conventional office measurements.

**Methods:**

Ambulatory patients with cirrhosis underwent office blood pressure and heart rate measurements, 24-hour ambulatory blood pressure monitoring and blood laboratory tests.

**Results:**

Fifty-one patients (32 men, mean age 57.4 ± 11.3 years) completed the study. Twenty six patients had compensated liver cirrhosis (group A) and 25 patients had more advanced liver disease (group B). Group A and B patients differed significantly both in ambulatory asleep diastolic blood pressure (p < 0.05) and office diastolic blood pressure (p < 0.01), which were lower in more advanced liver disease. Office blood pressure and heart rate correlations were similar to or even stronger than ambulatory ones. Ambulatory blood pressure and heart rate awake-asleep variation (dipping) showed a relatively flat pattern as markers of liver dysfunction were deteriorating. The strongest correlations were found with both ambulatory and office heart rate, which increased as indicators of severity of liver disease were worsening.

**Conclusions:**

Heart rate seems to be a more reliable marker of ongoing liver dysfunction than blood pressure. Evaluation of blood pressure and heart rate with 24-hour ambulatory measurement does not seem to offer more information than conventional office measurements.

## Background

Hepatic cirrhosis is accompanied by alterations of systemic circulation, such as reduced peripheral vascular resistance, low or low-normal blood pressure (BP) and increased heart rate (HR), stroke volume and cardiac output at rest, a situation which is called "hyperdynamic circulation". These alterations deteriorate further with the aggravation of hepatocellular failure [1-5]. It has been shown that these changes are associated with overall survival of cirrhotic patients [6,7]. Reduced vascular resistance in cirrhotic patients has been attributed to several circulating vasodilators [8-15]. Peripheral vasodilatation in cirrhosis leads to reduced "effective" arterial blood volume and activation of counteracting vasoconstrictor systems such as renin-angiotensin-aldosterone system, sympathetic nervous system and hypothalamic-pituitary system (vasopressin secretion) [3]. Consequently, urine water and sodium excretion is reduced and total blood volume is expanded.

Only two published trials have compared non-invasive 24-hour ambulatory blood pressure (ABP) parameters of cirrhotic patients with those of healthy controls and investigated their correlation with markers of severity of liver disease [16,17]. It has been suggested that 24-hour ABP monitoring, compared with conventional office BP measurements, has higher reproducibility and stronger correlation with target organ damage and total cardiovascular risk in hypertensive patients [18-25]. We hypothesized that, as long as office BP and HR are related to the severity of liver damage, a detailed assessment of the BP profile, as provided by 24-hour ABP measurement, would be proved a reliable index of liver dysfunction.

In this study, ambulatory cirrhotic patients underwent 24-hour ABP monitoring, as well as routine office BP and HR measurements. Additionally, biochemical and hormonal markers of liver function were tested. Patients were divided into two groups, those with Child A (group A) and those with Child B and C liver disease (group B). BP and HR parameters were compared between the two groups and statistical correlations between those parameters and aforementioned markers of liver function and portal hypertension were studied, in order to assess whether non-invasive 24-hour measurements are more closely associated with markers of liver disease severity than the conventional office measurements.

## Methods

### Subjects

This cross-sectional study included ambulatory patients with liver cirrhosis irrespective of aetiology, sex or age (> 18 years). Patients with known history of arterial hypertension, treated or untreated, heart failure, cardiac cirrhosis, bedridden, with clinical signs of hepatic encephalopathy, active infection or hemorrhage within the last 4 weeks before the study, active alcohol consumption or symptoms of withdrawal and patients with serum creatinine >133 μmol/L or serum Na <130 mmol/L on two consecutive occasions, were excluded. Twenty six patients with Child A cirrhosis formed the group A. Twenty one Child B and 4 Child C patients formed the group B. The latter 4 patients were borderline for Child C. All subjects gave written informed consent before undergoing the procedures of the study. The study protocol was approved by Local Ethics Committee.

### General information

Study procedures were performed at a Day Clinic of a University Hospital. All participants gave routine medical history and underwent physical examination. The following characteristics were recorded for each subject: age, sex, body height and weight, history of diabetes mellitus, smoking habits (smokers, ex-smokers, non-smokers), concomitant medical treatment, peripheral oedema or ascites, confirmed by physical examination or recent imaging examination and aetiology of cirrhosis. Child score and MELD score [26] were calculated. Diuretics, b-blockers or other drugs affecting BP, when used, were stopped 7 days before and during the study. No specific instructions for salt intake were given. Ten of the patients included in the study were treated with b-blockers. The indication of b-blockers was first variceal bleeding prophylaxis. All participants who stopped temporarily b-blockers had low risk of variceal bleeding (grade A esophageal varices and no history of variceal bleeding). B-blockers were withdrawn for 8 days (7 days wash-out plus one day for the study procedures). The consultant hepatologists (S.P.D and A.A) considered that there was no additional risk for the above patients from 8-day b-blocker withdrawal. To demonstrate the safety of this procedure, information about mortality and morbidity, including variceal bleeding and hospital admission due to liver disease, was retrospectively reviewed for all participants 12 months after their inclusion into the study. This information was obtained from outpatient clinic files or by telephone contact.

### Office BP and HR measurements

Office BP and HR were measured on two consecutive morning visits, by physicians trained according to the British Hypertension Society Protocol, using a mercury sphygmomanometer [27]. Measurements were performed on the left arm, with the subject sitting for at least 5 min. Cuffs with inflatable bladders encircling >80% of the arm circumference of each individual, were used. The 1^st ^Korotkoff sound was recorded as systolic blood pressure (SBP) and the 5^th ^as diastolic blood pressure (DBP). Three measurements were taken for each subject with one min interval. HR was measured once after the 1^st ^BP measurement, with palpation of the radial pulse for 30 sec.

### Ambulatory BP and HR measurements

Ambulatory BP and HR were monitored for 24 hours, on a routine working day, using validated oscillometric devices Spacelabs 90207 or 90217 (Spacelabs Inc, Redmond, WA, USA, with bladder size 23 × 12 cm or 30 × 14 cm, where appropriate) [28]. Measurements were taken at 20-min intervals during the whole 24-hour period. Patients were instructed to follow their usual daily activities, but to remain still with their forearm extended during each BP reading and to keep a brief timetable of the hours they stayed in bed. ABP measurements with <20 valid readings during the day and/or <10 during the night had to be repeated. The accuracy of each ABP device was tested before applying to each subject against a mercury column (Y-connection). Three successive measurements were made, in order to ensure that the difference between oscillometric and stethoscopic reading was not >10 mmHg in all 3 SBP or DBP readings. In that case the device was substituted by another one and tested again.

### Laboratory tests

Blood samples for international normalized ratio (INR), total bilirubin, alanine and aspartate aminotransferases (ALT, AST), albumin, sodium, creatinine, renin and aldosterone were taken with the subject sitting on a chair for at least 10 min, fasting for 8 to 12 hours and after he/she had been awake and active for at least one hour. Samples were drawn either on the 1^st ^day of the study, after medical history was obtained, physical examination was performed and office BP measurements were made and before applying the ABP device, or on the 2^nd ^day, after withdrawing the ABP device and performing the office BP measurements. Renin and aldosterone concentration was measured after serum specimens were centrifuged and frozen at below -20°C for <6 months. Routine laboratory methods were used for INR and biochemistry tests. Serum renin and aldosterone concentration was measured with Nichols Advantage® Direct Renin and Nichols Advantage® Aldosterone chemiluminescense methods (normal values in upright position 3.3-41 μIU/ml for renin and 3-34 ng/dl for aldosterone).

### Statistical Analysis

For each office visit average sitting SBP, DBP and HR were included in the analysis. Ambulatory SBP, DBP and HR readings were averaged to 24-hour, awake and asleep values, according to each individual subject's sleeping hours. Logarithmic transformation was performed in not normally distributed data (total bilirubin, ALT, AST, renin and aldosterone). T-tests were used to evaluate the difference of the mean of quantitative variables among two groups. X^2 ^test was used to evaluate frequency differences of qualitative variables among two groups. Statistical correlations of office and ambulatory BP and HR measurements with each of the following markers, i.e. Child score, MELD score, total bilirubin, albumin, INR, ALT, AST, renin and aldosterone, were studied and Pearson correlation coefficients were calculated. Quantitative variables are presented as mean ± SD. Statistical software MINITAB INC (release 13.31, State College, Pennsylvania, USA) was used for all statistical procedures. A p value of < 0.05 was considered statistically significant.

## Results

### Patients' characteristics

From November 2003 until May 2006 60 consecutive patients were recruited. Five refused to participate, 3 were excluded due to history of hypertension, and one due to poor general condition (not fully ambulatory). Fifty-one patients completed the study, (32 men), with mean age 57.4 ± 11.3 (SD) years. The cause of cirrhosis was alcohol abuse, viral hepatitis and miscellaneous in 23, 20 and 8 patients respectively. Twenty six, 21 and 4 patients were Child A, B and C respectively. Child B and C patients were analyzed together (group B). Mean Child score was 6.7 in all patients, 5.3 in group A and 8.2 in group B. Mean MELD score was 11.7, 8.9 and 14.7, respectively. Demographic data and other characteristics of the participants are presented in Table 1.

**Table 1:** Characteristics of study population according to the severity of liver disease

	Group A	Group B	All	p
**N**	26	25	51	
**Male (%)**	50	76	63	0.05
**Age (years)**	56.7 ± 13.0	58.1 ± 9.4	57.4 ± 11.3	NS
**BMI (kg/m^2^)**	25.9 ± 3.8	27.2 ± 4.8	26.5 ± 4.3	NS
**Diabetes (%)**	11.5	8	9.8	NS
**Active smokers (%)**	38.5	52	45.1	NS
**Alcoholic cirrhosis (%)**	30.8	60	45.1	< 0.05
**Hepatitis B (%)**	26.9	16	21.6	NS
**Hepatitis C (%)**	23.1	12	17.6	NS
**PBC (%)**	11.5	0	5.9	NS
**Autoimmune hepatitis(%)**	3.8	4	3.9	NS
**Steatohepatitis (%)**	3.8	4	3.9	NS
**Cryptogenic cirrhosis(%)**	0	4	2	NS
**Ascites (%)**	0	52	25.5	< 0.001
**Peripheral oedema (%)**	0	12	5.9	NS
**Total bilirubin (μmol/L)**	18.8 ± 8.5	51.3 ± 47.9	34.2 ± 37.6	< 0.001
**Albumin (g/L)**	40.0 ± 5.0	33.0 ± 4.0	37.0 ± 5.0	< 0.001
**INR**	1.2 ± 0.1	1.5 ± 0.3	1.3 ± 0.3	< 0.001
**ALT (IU/l)**	44.5 ± 34.7	55.6 ± 56.4	49.9 ± 46.5	NS
**AST (IU/l)**	52.7 ± 38.4	84.3 ± 69.3	68.2 ± 57.4	< 0.01
**Renin (μIU/ml)**	27.5 ± 29.7	90.3 ± 161.9	57.7 ± 117.3	NS
**Aldosterone (ng/dl)**	11.7 ± 10.1	41.9 ± 85.2	26.2 ± 60.8	NS

### Comparison of office and ambulatory BP and HR parameters between group A and group B patients

Ambulatory and office BP and HR values are presented in Table 2. The only statistically significant difference between group A and group B was in ambulatory asleep DBP (p < 0.05) and in office DBP. As expected, established markers of liver function (total serum bilirubin, INR, serum albumin) differed significantly between the two groups (Table 1).

**Table 2:** Comparison of office and ambulatory blood pressure (mmHg) and heart rate (beats per min) in group A versus group B patients

		Group A	Group B	All	p
**Office** **Visit 1**	SBP	134.9 ± 17.4	127.4 ± 16.7	131.2 ± 17.3	NS
	DBP	81.9 ± 11.3	73.8 ± 7.6	77.9 ± 10.4	< 0.01
	HR	72.7 ± 11.6	74.4 ± 10.6	73.5 ± 11.1	NS
**Office** **Visit 2**	SBP	130.6 ± 16.8	125.3 ± 15.0	128.0 ± 16.0	NS
	DBP	79.8 ± 10.5	72.5 ± 8.5	76.1 ± 10.2	0.01
	HR	73.8 ± 10.1	78.4 ± 13.6	76.1 ± 12.1	NS
**24-hour ambulatory**	SBP	121.3 ± 14.3	121.7 ± 10.1	121.5 ± 12.3	NS
	DBP	72.9 ± 8.8	69.6 ± 5.4	71.3 ± 7.4	NS
	HR	73.8 ± 10.7	77.3 ± 10.6	75.5 ± 10.7	NS
**Awake ambulatory**	SBP	127.1 ± 14.1	128.4 ± 12.2	127.7 ± 13.0	NS
	DBP	77.8 ± 8.7	74.6 ± 5.2	76.2 ± 7.3	NS
	HR	78.1 ± 11.0	79.9 ± 10.6	79.0 ± 10.7	NS
**Asleep ambulatory**	SBP	114.0 ± 14.9	111.5 ± 10.0	112.8 ± 12.7	NS
	DBP	66.8 ± 9.7	61.8 ± 7.0	64.4 ± 8.8	< 0.05
	HR	68.0 ± 12.0	72.8 ± 10.7	70.3 ± 11.6	NS
**Awake-asleep difference**	SBP	13.1 ± 6.1	17.0 ± 8.9	15.0 ± 7.8	NS
	DBP	11.0 ± 4.7	12.8 ± 6.0	11.9 ± 5.4	NS
	HR	10.1 ± 7.6	7.1 ± 5.8	8.6 ± 6.9	NS

SBP, systolic blood pressure; DBP, diastolic blood pressure; HR, heart rate

### Correlations between office and ambulatory blood pressure and laboratory markers

The relationship between office and ambulatory BP and laboratory biochemical and hormonal markers is summarized in Table 3. Awake ambulatory SBP correlated negatively with serum renin. 24-hour and asleep ambulatory SBP gave no significant correlations, either with Child and MELD scores, or with laboratory parameters. On the other hand, office SBP measurements showed stronger correlations, negative with Child score, INR, serum renin and aldosterone concentration and positive with serum albumin. Statistically significant positive correlations were found between ambulatory DBP (24-hour, awake and asleep) and serum albumin. Office DBP measurements on the other hand, showed stronger correlations, negative with Child and MELD scores, total serum bilirubin and INR and positive with serum albumin.

**Table 3:** Correlations of office and ambulatory blood pressure with clinical and laboratory markers of liver disease.

	Office SBP/DBP Visit 1	Office SBP/DBP Visit 2	24-hour ambulatory SBP/DBP	Awake ambulatory SBP/DBP	Asleep ambulatory SBP/DBP
**Child score**	-0.33*/-0.39**	-0.25/-0.36**	-0.04/-0.17	-0.05/-0.21	-0.05/-0.15
**MELD score**	-0.25/-0.30*	-0.20/-0.29*	0.03/-0.06	0.01/-0.09	0.02/-0.06
**T. Bilirubin**	-0.18/-0.28*	-0.11/-0.26	0.09/-0.04	0.10/-0.05	0.04/-0.06
**Albumin**	0.34 */0.51***	0.21/0.44***	0.07/0.33**	0.07/0.38**	0.11/0.32*
**INR**	-0.24/-0.23	-0.31*/-0.30*	-0.03/-0.07	-0.03/-0.08	-0.05/-0.08
**ALT**	-0.04/-0.16	-0.13/-0.13	-0.17/-0.19	-0.13/-0.16	-0.20/-0.21
**AST**	-0.03/-0.23	-0.08/-0.21	-0.08/-0.20	-0.03/-0.18	-0.16/-0.25
**Renin**	-0.36**/-0.09	-0.29*/-0.07	-0.21/0.03	-0.30*/-0.05	-0.11/0.09
**Aldosterone**	-0.30*/-0.12	-0.25/-0.08	-0.07/0.10	-0.16/-0.01	0.05/0.19

(Correlation coefficients r)

SBP, systolic blood pressure; DBP, diastolic blood pressure;

*, p < 0.05; **, p < 0.01; ***, p < 0.001

### Correlations between office and ambulatory HR and laboratory parameters

**Figure 1:**
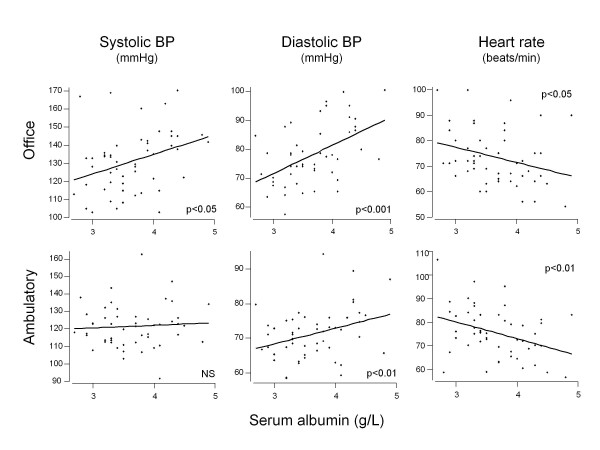
**Correlations between serum albumin and blood pressure (BP) and heart rate measurements in the office (visit 1) and with 24-hour ambulatory monitoring**.

Both office and ambulatory HR measurements showed stronger correlations than BP parameters, positive with Child and MELD scores, total serum bilirubin, INR, renin and aldosterone and negative with serum albumin (Table 4). Correlations between serum albumin and BP and HR measurements in the office (visit 1) and with 24-hour ambulatory monitoring are presented as scatter plots in Figure 1.

**Table 4:** Correlations of office and ambulatory heart rate with clinical and laboratory markers of liver disease.

	Office HR Visit 1	Office HR Visit 2	24-hour ambulatory HR	Awake ambulatory HR	Asleep ambulatory HR
**Child score**	0.27	0.43**	0.32*	0.24	0.35*
**MELD score**	0.32*	0.40**	0.36**	0.28*	0.40**
**T. Bilirubin**	0.34*	0.40**	0.35**	0.28*	0.40**
**Albumin**	- 0.29*	- 0.44***	- 0.36**	-0.25	- 0.45***
**INR**	0.31*	0.35*	0.36**	0.31*	0.33**
**ALT**	0.10	0.09	0.01	0.02	0
**AST**	0.18	0.21	0.17	0.14	0.20
**Renin**	0.35*	0.56***	0.49***	0.48***	0.43**
**Aldosterone**	0.41**	0.48***	0.44***	0.40**	0.44***

(Correlation coefficients r)

HR, heart rate; *, p < 0.05; **, p < 0.01; ***, p < 0.001

### BP and HR "dipping" and laboratory parameters

Awake-asleep differences in ambulatory measurements (dipping) revealed a few correlations with laboratory parameters. Correlations were negative between SBP dipping, renin and aldosterone concentration (-0.33 and -0.35, p < 0.05 respectively), negative between DBP dipping and aldosterone concentration (-0.32, p < 0.05), positive between HR dipping and albumin concentration (0.35, p < 0.05) and negative between HR dipping and Child score (-0.22, p < 0.05). Comparisons between group A and group B regarding BP and HR awake-asleep dipping revealed contradictory and not statistically significant differences (Table 2).

Retrospective assessment of patients' health status 12 months after their participation into the study revealed information for 50 of the 51 study participants (one was lost to follow-up). Two patients have died, one due to hepatocellular carcinoma and another due to progressive liver failure. None of the abovementioned 3 patients was on b-blocker therapy before the study. The rest 48 patients were alive one year after study inclusion, with no further complications related to liver disease.

## Discussion

This is the first study that evaluated the 24-hour BP and HR profile in relation to markers of liver function in patients with cirrhosis in fully ambulatory conditions. Two previous studies have assessed the ABP profile in patients with alcoholic cirrhosis, of whom about half were at Child C stage. Both of these studies have been conducted in hospitalized patients and continued diuretic treatment, which have probably influenced the study findings. These studies showed that ABP is lower in patients than in controls, especially during the day. Awake and asleep HR was higher in cirrhotic patients and awake-asleep variation was lower than in controls. BP and HR parameters correlated with indices of liver function. Among cirrhotic patients, only the difference in DBP between Child A and C reached statistical significance [16,17].

This study included cirrhotic, non-hospitalized, patients, of whom only 4 marginally fulfilled Child stage C criteria. Therefore, the differences in BP and HR between study groups had to be striking, in order to be revealed. This study intended to investigate the relationship of the 24-hour ambulatory BP and HR with markers of liver dysfunction. Therefore, only fully ambulatory patients were included in whom a few days withdrawal of drug treatment (diuretics and b-blockers) was acceptable. On the other hand, severely diseased patients in whom the diurnal variation of these hemodynamic parameters was distorted due to limited physical activity, which is outside the standards of ABP monitoring [25] and in whom drug treatment could not be withdrawn, were excluded.

A consistent relationship between both the degree of BP reduction and tachycardia and the severity of hepatic dysfunction was shown in the present study. These findings are in line with previous observations showing that BP is reduced and HR was elevated in advanced liver disease [1-5]. DBP in both office visits and asleep ambulatory DBP were significantly lower in the group with more advanced liver disease. HR did not differ significantly between group A and B patients (Table 2). It is worth noting though, that among all hemodynamic parameters, HR exhibited the strongest association with markers of liver disease severity (Tables 3 and 4). Because the sample size is relatively small, the main analysis was performed by treating the data as continuous rather than categorical variables, which allows the assessment of associations. The correlation factors are relatively low and according to the r^2 ^value for the association between HR of office visit 2 and serum renin, only 31% of the variation of HR could be explained by the renin variation (Table 4). Attempting to explain the finding that HR was more closely related to markers of liver function than other hemodynamic parameters, we presume that homeostatic mechanisms, such as renin-angiotensin-aldosterone system and sympathetic nervous system, counteracting the reduction of effective arterial blood volume in cirrhotic patients, attempt to "correct" BP towards normal levels, in the expense of HR elevation [29-31]. Another assumption could be that elevated cardiac output, which has been observed in cirrhotic patients, is achieved more effectively through elevation of HR and less through elevation of stroke volume, because of the compromised capacity of the left ventricle to raise stroke volume (cirrhotic cardiomyopathy) [32-34]. It is noteworthy that serum albumin is related with all hemodynamic parameters studied (Tables 3 and 4) suggesting that, apart from peripheral vasodilatation, plasma oncotic pressure reduction remains an important determinant of hyperdynamic circulation in cirrhosis.

Although 24-hour ambulatory values are considered more reliable than office measurements in the evaluation of BP and HR in hypertensive patients, this does not seem to be the case in the setting of cirrhotic patients [18-25]. Indeed, the effect of differences in daytime activity during 24 h ambulatory monitoring is offset by the larger number of readings and the fact that these are taken in routine daily conditions, resulting thereby to superior reproducibility of ambulatory compared to office BP measurements. We hypothesized that because hemodynamic parameters such as low office BP and increased HR are related to the severity of liver damage, a detailed evaluation of the BP profile, as provided by 24-hour ambulatory BP measurement, would be proved a more reliable index of liver dysfunction than the conventional hemodynamic assessment (office BP and HR). The unexpected finding that office measurements were not inferior to ambulatory measurements, could be attributed to the meticulous and according to relevant guidelines procedure of office BP and HR measurements, in the setting of a hypertension research unit. Even if 24-hour ABP is proved accurate and valuable in hypertensive patients [18-25], its usefulness in the population of cirrhotic patients is therefore not well documented.

Another interesting finding is the reduced awake-asleep variation of BP and HR in patients with more severe portal hypertension, as suggested by higher renin and aldosterone levels. Reduced awake-asleep variation of BP and HR has also been demonstrated in patients with more severe hepatocellular dysfunction, as suggested by lower albumin levels. The presence of reduced awake-asleep variation in more advanced liver disease has also been reported by previous studies [16,17,35,36]. Since a major determinant of awake-asleep variation of BP and HR is physical activity [37], it could be hypothesized that cirrhotic patients have less physical activity than normal subjects, even though patients in a poor condition have been excluded in this study. Reduced awake-asleep variation of BP and HR might also be attributed to impaired autonomic nervous activity, which has been reported in cirrhotic patients [38-40], leading to vascular hyporeactivity. Cirrhotic patients have been reported to exhibit an almost unaltered cardiac output and a small alteration in vascular resistance from daytime to nighttime [16,17]. These findings are consistent with a general circulatory hyporeactivity in cirrhosis, comprising a blunt response to stimuli that normally influence circulation [32-34]. A limitation of the present study concerning this point is that alcoholic cirrhosis was significantly more frequent among group B patients (Table 1).

This was a cross-sectional study investigating associations and was not designed to assess hard end-points. Moreover, the study sample is too small to allow conclusions in relation to outcome. Further research is needed, in order to investigate the association between hemodynamic parameters and hard endpoints such as survival and hospitalization.

## Conclusions

This study showed that HR, measured either with ABP monitoring or conventional office measurement, seems to be a more reliable marker of ongoing liver dysfunction than BP. Given that normally the range of HR is wide, it is not possible to define a threshold that might indicate severe liver insufficiency. An abnormal pattern of 24-hour BP and HR daily variation was observed probably due to lack of regulation of the aforementioned hemodynamic parameters from daytime to nighttime. This abnormal diurnal pattern was evident in advanced liver disease. In conclusion, these data do not support the use of 24-hour ambulatory BP and HR as a more accurate method than office measurements for the evaluation of the severity of liver insufficiency.

## Competing interests

The authors declare that they have no competing interests.

## Authors' contributions

GSS had the main idea for this study and prepared the first draft of the protocol, supervised the study execution and data analysis and contributed to data interpretation and manuscript preparation. DGT collected the study data, prepared the electronic spreadsheet, performed the statistical analysis and wrote the first draft of the manuscript. SPD was involved in study design and subjects' recruitment and contributed to data interpretation. AA was involved in subjects' recruitment and contributed to data interpretation and preparation of the manuscript. All authors have read and approved the final manuscript.

## Pre-publication history

The pre-publication history for this paper can be accessed here:

http://www.biomedcentral.com/1471-230X/10/143/prepub
